# The Meiotic Spindle, Mechanisms of Chromosome Segregation and Aneuploidy in Aging Human Oocytes: An Update Review

**DOI:** 10.3390/medicina62091810

**Published:** 2026-09-20

**Authors:** Romualdo Sciorio, Gyongyver Teglas, Luca Tramontano, Laszlo Nanassy, Laura Girardi, Steven Fleming

**Affiliations:** 1 Vitrolife-Group, 421 32 Västra Frölunda, Sweden; 2 Department of Reproductive Medicine and Gynecological Endocrinology, Lucerne Cantonal Hospital, 6004 Lucerne, Switzerland; 3 Endometriosis and Fertility Center, Hôpital de La Tour, 1217 Meyrin, Switzerland; 4 Departement de Gynecologie-Obstetrique, Reseau Hospitalier Neuchâtelois, 2000 Neuchâtel, Switzerland; 5 Division of Gynecological Endocrinology and Reproductive Medicine, Department of Obstetrics and Gynecology, Medical University of Vienna, 1090 Vienna, Austria; 6 Igenomix Italy, Reproductive Genetics, 36063 Marostica, Italy; 7 Discipline of Anatomy & Histology, School of Medical Sciences, University of Sydney, Sydney, NSW 2006, Australia

**Keywords:** human oocyte, meiotic spindle, chromosome segregation, aneuploidy, maternal age, assisted reproductive technology (ART)

## Abstract

The meiotic spindle is essential for accurate chromosome segregation during oocyte maturation and fertilization, and its alteration might be considered a major contributor to age-related aneuploidy and reproductive failure. Spindle formation is inherently vulnerable to errors and contributes to the unusually high frequency of chromosome segregation defects observed in human eggs. Human oocytes are particularly prone to aneuploidy; the presence of an abnormal chromosome number, which represents one of the leading causes of infertility, recurrent pregnancy loss, implantation failure, and congenital disorders in neonates. Previous studies have reported a U-shaped relationship between maternal age and aneuploidy rates, with relatively higher rates observed in both very young females and in women of advanced maternal age. Conversely, aneuploidy rates appear comparatively lower during the intervening reproductive-age period, particularly between approximately 25 and 35 years of age. Because most aneuploid embryos fail to develop successfully after fertilization, chromosome segregation errors in oocytes have profound consequences for female reproductive success. Aneuploidy primarily arises from defects during meiotic chromosome segregation. Faithful chromosome segregation depends on the accurate assembly and function of the meiotic spindle, a dynamic microtubule-based structure that aligns and separates homologous chromosomes during meiosis I and sister chromatids during meiosis II. Growing evidence indicates that chromosome mis-segregation results from the combined effects of impaired sister chromatid cohesion, altered kinetochore architecture, defective microtubule dynamics, weakened spindle assembly checkpoint activity, and age-related metabolic decline. Together, these defects compromise spindle integrity and reduce the fidelity of chromosome separation. Furthermore, the intrinsic instability of the acentrosomal spindle increases the likelihood of incorrect microtubule–kinetochore attachments, a defect that becomes more prevalent with maternal aging due to progressive deterioration of chromosome organization and kinetochore function. This review summarizes the current understanding of the molecular mechanisms regulating meiotic spindle assembly and function in human oocytes and examines how their disruption contributes to chromosome segregation errors and embryonic aneuploidy. We discuss the interplay between spindle abnormalities and upstream cellular defects, distinguish meiotic from post-zygotic origins of chromosomal abnormalities, and evaluate the clinical significance of spindle assessment in assisted reproductive technologies. Finally, we critically examine emerging strategies aimed at preserving chromosome segregation fidelity, including approaches targeting mitochondrial function and spindle regulation, while distinguishing interventions supported by mechanistic evidence from those that remain preclinical or speculative.

## 1. Introduction

Aneuploidy in human oocytes is a major biological contributor to reduced female fertility, early pregnancy loss, and chromosomally abnormal conception [1]. Its clinical relevance is evident in assisted reproductive technologies (ART), where increasing maternal age is associated with reduced oocyte competence, diminished embryonic developmental potential, and a higher proportion of chromosomally abnormal embryos [2]. However, meiotic chromosome errors are not restricted to advanced maternal age (AMA). Human oocytes from younger women also display a non-negligible baseline frequency of aneuploidy [3], suggesting that female meiosis is intrinsically vulnerable to error [4]. Embryonic chromosome abnormalities can originate during parental meiosis or arise post-zygotically through mitotic chromosome-segregation errors during the early stage of embryo development [5]. Post-zygotic errors are particularly relevant to embryonic mosaicism and represent a biologically distinct process from meiotic aneuploidy in the oocyte [6]. Accurate chromosome segregation during oocyte meiosis is strictly dependent upon the meiotic spindle (MS) apparatus, a dynamic microtubule-based structure that captures, aligns and separates chromosomes during the first and second meiotic divisions [7,8]. Human oocytes assemble their spindles without canonical centrosomes and lack the prominent acentriolar microtubule-organizing centres (aMTOCs) found in mouse oocytes. Instead, MS formation relies predominantly on chromosome-mediated microtubule nucleation, self-organization, and progressive pole focusing [9,10,11]. This atypical pathway requires approximately 16 h, markedly longer than the 3–5 h reported for mouse oocytes and considerably longer than typical MS assembly [9]. During this extended interval, transient multipolarity, unstable spindle poles, and delayed chromosome positioning may occur, prolonging the window during which erroneous kinetochore-microtubule (K-MT) attachments can form and persist [9,10,11]. In particular, merotelic attachments may persist despite apparently normal chromosome alignment, generating lagging chromosomes during anaphase [12]. In human oocytes, the prolonged, error-prone spindle-assembly pathway may induce the formation and persistence of these attachment errors [9]. Maternal ageing further increases this vulnerability through the progressive deterioration of chromosome cohesion, bivalent architecture, kinetochore geometry, spindle dynamics, and cell-cycle surveillance [2,13]. Cohesion weakening increases sister kinetochore separation and destabilizes bivalent structure, thereby promoting attachment configurations that are inappropriate for meiosis I [13,14,15]. In parallel, the spindle assembly checkpoint (SAC) is relatively permissive in oocytes and may delay, rather than completely prevent, anaphase in the presence of residual attachment errors [16,17]. Overall, the relationship between spindle abnormalities and aneuploidy is multifactorial and cannot be explained by a single causal pathway. Both may arise from shared upstream defects, although specific perturbations of spindle assembly or microtubule dynamics can also directly promote chromosome mis-segregation. This review examines the mechanisms regulating human MS assembly and how maternal age, chromosome architecture, checkpoint regulation, and metabolic homeostasis collectively compromise the fidelity of meiotic chromosome segregation, with consequences for embryonic chromosomal instability, embryo development, and pregnancy outcomes in ART.

## 2. Normal Meiotic Spindle Assembly and Chromosome Segregation

**Figure 1:**
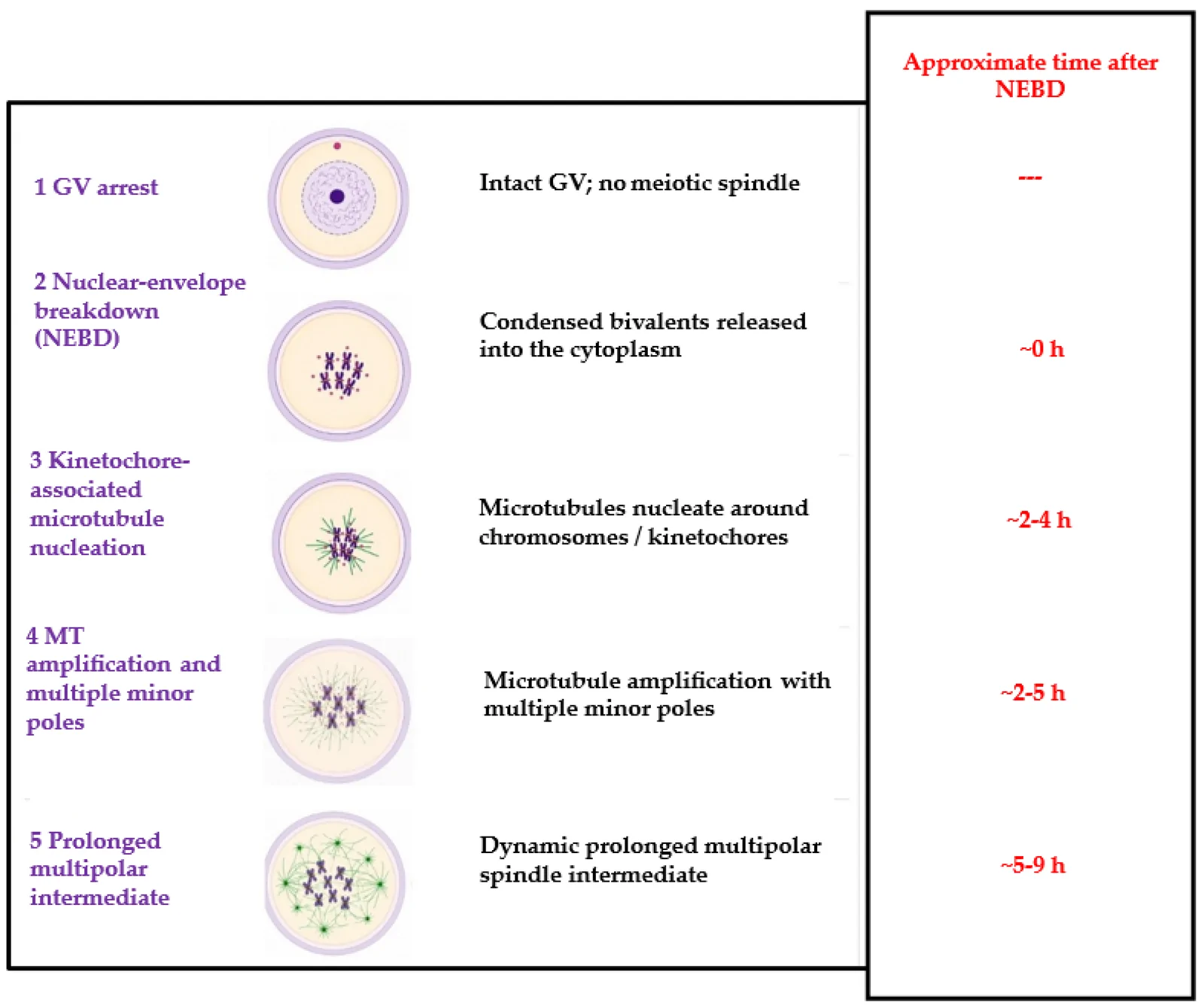

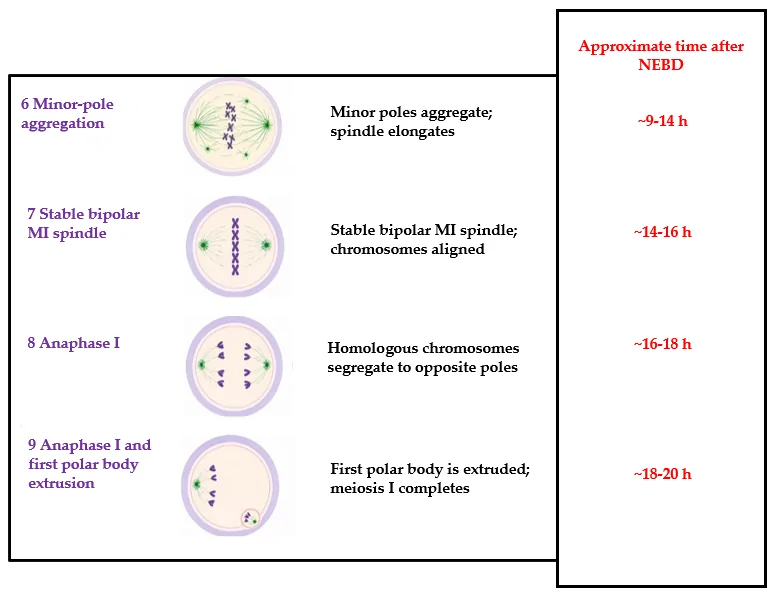
Temporal sequence of meiotic spindle assembly in human oocytes. Time refers to time following NEBD. GV: germinal vesicle; MI: metaphase 1; MT: microtubule; NEBD: nuclear-envelope breakdown.

**Figure 2:**
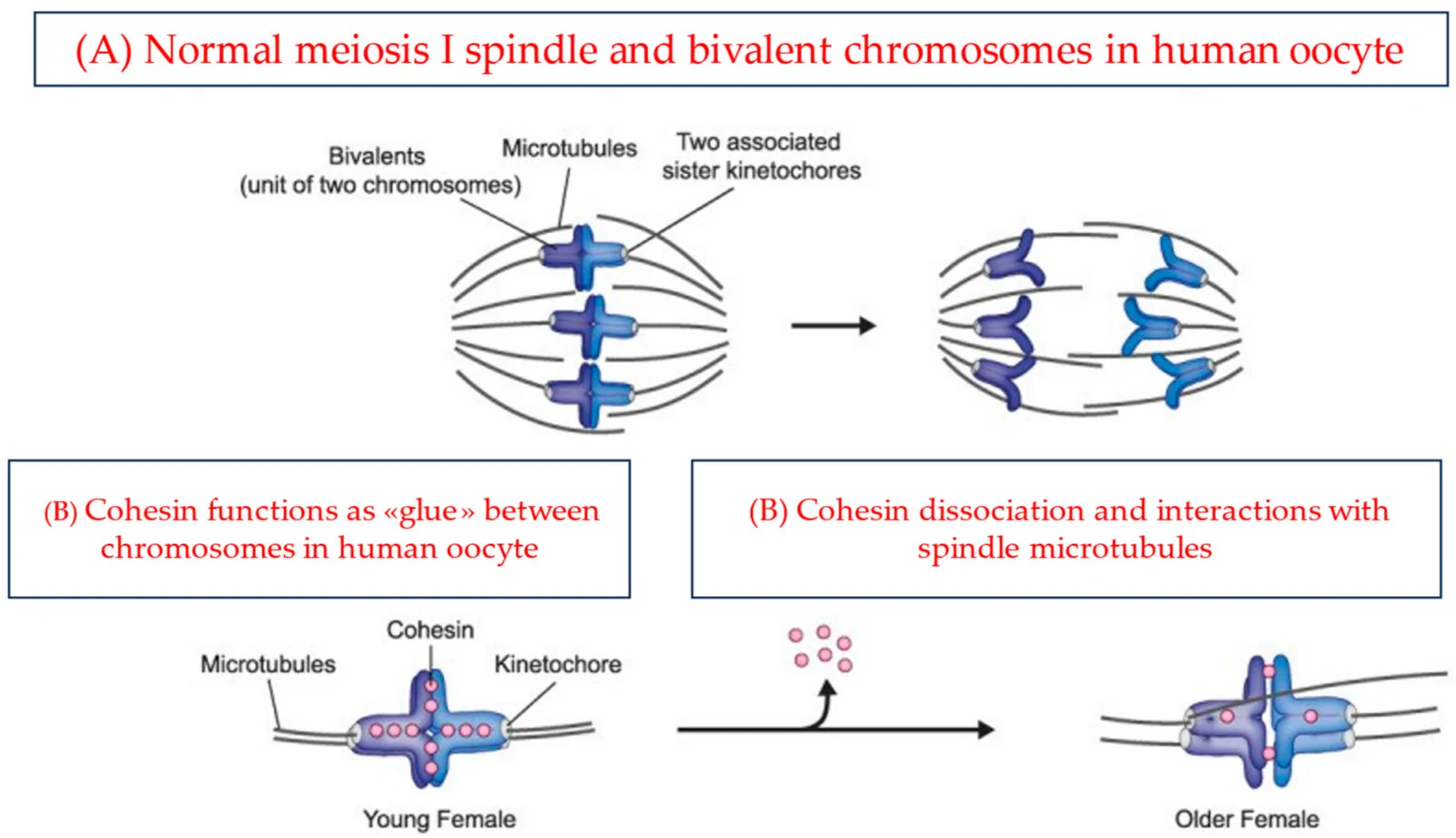
Structural changes and altered chromosome alignment during meiosis I (MI). (**A**) During MI, bivalents align at the metaphase I plate, with spindle microtubules interacting with sister kinetochores to ensure accurate chromosome segregation. (**B**) Age-related loss of cohesin weakens cohesion between sister chromatids and can disrupt chromosome alignment and spindle–kinetochore interactions, increasing the risk of meiotic chromosome mis-segregation.

Accurate chromosome segregation during female meiosis requires the formation of a bipolar spindle that captures, aligns, and segregates chromosomes within a highly specialized cellular environment [7,8,18]. Unlike somatic cells, mammalian oocytes assemble meiotic spindles without canonical centriole-containing centrosomes (Table 1). However, the mechanisms supporting acentrosomal spindle assembly differ substantially between species: mouse oocytes contain prominent aMTOCs that contribute to microtubule nucleation and pole formation, whereas human oocytes lack comparable dominant MTOCs and rely more heavily on chromosome-mediated spindle assembly [9,11,19]. Bovine oocytes appear to share some of these human-like features and may therefore provide an informative animal model for studying aspects of human spindle organization [11]. Following nuclear-envelope breakdown, microtubules formed around chromosomes are progressively organized into a bipolar spindle through the coordinated activity of chromosome-derived signals, motor proteins, and microtubule-associated factors [7,8]. More generally, studies in mitotic systems have shown that temporal coordination of distinct microtubule-assembly pathways is important for robust bipolar spindle formation, providing a conceptual framework to understand why delayed or poorly coordinated spindle self-organization may increase the risk of abnormal geometry in human oocytes [20]. During prometaphase I, bivalents are captured by spindle microtubules through their kinetochores. Stable kinetochore fibers are progressively established as correct attachment geometry and attachment tension develop [8,18]. A defining feature of meiosis I is the co-orientation of sister kinetochores towards the same spindle pole, allowing homologous chromosomes to segregate during anaphase I while sister chromatids remain together [7,16,17,18]. Cohesin complexes, together with chiasmata, preserve bivalent integrity until homologues separate. Weakening of cohesion can alter sister-kinetochore geometry, destabilize bivalents and permit attachment configurations that are inappropriate for meiosis I [13,14]. Correct K-MT attachment requires active error correction. Aurora kinases destabilize weak or improperly tensioned attachments by phosphorylating kinetochore substrates, allowing erroneous interactions to be released and re-established. Aurora B and Aurora C kinases are particularly important during mammalian oocyte meiosis, where they regulate chromosome alignment, error correction, and checkpoint signaling [21,22]. The SAC provides an additional safeguard by delaying anaphase until chromosomes are correctly attached to the spindle. Unattached or improperly attached kinetochores recruit checkpoint proteins, including MPS1, BUB1, BUBR1, MAD1 and MAD2, which restrain anaphase-promoting complex/cyclosome (APC/C) activity and prevent premature degradation of securin and cyclin B [8,16,23]. Securin inhibits separation, consequently maintaining cohesion until the appropriate stage of chromosome segregation. In human oocytes, BUB1 and BUBR1 localize to kinetochores in an MPS1-dependent manner, confirming that core SAC machinery is active during female meiosis [14]. However, the SAC in mammalian oocytes is relatively permissive. Rather than acting as an absolute block, it primarily delays meiotic progression when only a limited number of kinetochores are defective [16,18]. Consequently, some abnormal chromosome configurations may fail to generate a sufficiently strong checkpoint signal to prevent anaphase onset, despite remaining prone to chromosome segregation errors. For example, univalent or prematurely separated sister chromatids may establish bipolar K-MT attachments that appear normal because both kinetochores are occupied yet fail to maintain the meiosis-I-specific co-orientation required for accurate homologous chromosome segregation [13,14]. Thus, faithful meiotic chromosome segregation depends on the coordinated integration of acentrosomal spindle assembly, cohesion-dependent chromosome architecture, Aurora kinase (AURK) mediated error correction and SAC-dependent cell-cycle control. Figure 1 illustrates the temporal progression of MS formation in human oocytes, while Figure 2 shows the essential role of cohesion in correct chromosome alignment.

## 3. Spindle Abnormalities and Aneuploidy

Meiotic spindle defects can generate erroneous K-MT attachments and chromosome segregation errors, but they may also arise secondarily from altered chromosome architecture, cohesion loss, mitochondrial dysfunction, or defective cell-cycle regulation. The strength of the evidence therefore depends on whether spindle phenotype and chromosome outcome are assessed in the same oocyte or experimental system. Human in-vitro fertilization (IVF) studies based solely on spindle morphology provide indirect evidence, whereas live-cell imaging and experimental perturbation studies offer stronger mechanistic support for a causal relationship between spindle abnormalities and errors in correct chromosome separation [10,24,25].

### 3.1. Disorganized and Unstable Spindle Architecture

Spindle assembly in human oocytes is inherently susceptible to structural instability. In contrast to the rapid centrosome-dependent assembly of the mitotic spindle, MS formation relies on a chromosome-driven pathway that is slower and involves gradual organization of the spindle poles, often progressing through transient disordered or multipolar intermediates during meiosis I [9,10,11]. The extended duration of spindle assembly increases the time during which erroneous K–MT attachments can form and persist before chromosome segregation begins, thereby increasing the risk of errors during chromosome segregation. Studies demonstrate that spindle assembly is often delayed and dynamically unstable, allowing chromosomes to remain incompletely aligned, inducing abnormal microtubule interactions. They further show that spindle poles arise from fragmented microtubule-organising structures that require extensive clustering to generate a stable bipolar spindle [9,10,11]. Consequently, inefficient pole clustering or delayed spindle maturation may produce unstable or transiently multipolar spindle configurations that favour the persistence of incorrect K–MT attachments [9,10,11]. Evidence from animal and experimental models further supports a causal relationship between spindle abnormalities and chromosome segregation errors. Disruption of proteins involved in microtubule organisation, spindle-pole integrity, or K–MT coupling consistently results in spindle disorganisation, chromosome misalignment, and aneuploid oocytes. For example, perturbation of various components of the chromosomal passenger complex, including CENP-V, SPC24, or CDCA8, impairs spindle-associated chromosome interactions and compromises faithful chromosome segregation in both mouse and human oocytes [26,27,28]. Furthermore, live-imaging RNA interference (RNAi) screens have identified an extensive network of genes required for mammalian oocyte meiosis, highlighting that spindle integrity depends on the coordinated activity of multiple interacting regulators rather than on a single structural component [29].

### 3.2. Multipolarity and Abnormal Spindle Geometry

Transient multipolarity is a distinctive feature of MS assembly in human oocytes. Rather than forming directly as a stable bipolar structure, the spindle frequently progresses through transient multipolar intermediate states before the spindle poles become fully focused. During this dynamic assembly process, chromosomes may interact with microtubules originating from multiple directions, increasing the likelihood of erroneous K-MT attachments and abnormal chromosome separation [9,10,11]. Spindle geometry and chromosome architecture are closely interconnected, with defects in one often influencing the stability of the other. Broad, asymmetric, or poorly focused spindles can disrupt the spatial organisation of chromosomes and microtubules, whereas age-related weakening of chromosome cohesion alters kinetochore orientation and compromises the meiosis-I-specific co-orientation required for accurate homologous chromosome segregation [13,14]. Volumetric morphometric analyses indicate that spindle width is a useful parameter for assessing spindle scaling in mammalian oocytes. Nevertheless, abnormal spindle geometry alone should not be interpreted as definitive evidence of aneuploidy without direct assessment of chromosome segregation outcomes [30].

### 3.3. Spindle Displacement and Absence of Visible Spindles

Spindle positioning represents an additional determinant of a correct chromosome separation. In mouse oocytes, the central positioning of the meiosis I spindle provides sufficient time for proper K-MT attachment formation and error correction before the spindle migrates towards the cortex. Premature spindle proximity to the cortex increases CDC42-dependent microtubule tyrosination, compromises kinetochore-fibre stability, and promotes the persistence of erroneous K–MT attachments. These defects are associated with elevated aneuploidy rates, providing direct experimental evidence that abnormal spindle positioning can contribute to incorrect chromosome segregation [31]. This model demonstrates that a spindle can maintain an apparently normal bipolar morphology while remaining functionally compromised because its spatial position exposes it to cortical signals that modify microtubule dynamics and attachment stability. Although this mechanism has been established primarily in mouse oocytes, it provides an important conceptual framework for understanding how spindle displacement or altered spatial regulation may influence chromosome separation in human oocytes. In ART treatments, spindle organisation is commonly evaluated using polarised-light microscopy, which detects birefringent microtubule structures within the MS [10,32,33]. However, the absence of a visible spindle does not necessarily indicate structural failure, as it may reflect delayed spindle maturation, spindle orientation relative to the imaging axis, technical limitations, or reduced birefringence. Therefore, spindle non-visualisation should be interpreted cautiously and cannot be considered direct evidence of aneuploidy without corresponding chromosome-level analysis in the same oocyte or resulting embryo [10,24].

### 3.4. Chromosome Misalignment as a Bridge to Aneuploidy

Chromosome misalignment represents a critical mechanistic link between spindle dysfunction and oocyte aneuploidy. When spindle microtubules fail to establish stable and correctly oriented K-MT attachments, chromosomes may remain displaced from the metaphase plate, exhibit delayed alignment, or undergo unequal distribution during anaphase. Abnormal spindle-associated segregation patterns, such as tri-directional anaphases observed in human oocytes, provide striking examples of defective chromosome partitioning and can result in highly abnormal chromosomal complements [34]. Merotelic attachments, in which a single kinetochore simultaneously interacts with microtubules originating from both spindle poles, are particularly problematic because they may persist despite apparently normal chromosome alignment. Studies in aged mouse oocytes demonstrate that merotelic attachments during meiosis II promote sister chromatid segregation errors, highlighting how subtle attachment defects can significantly compromise ploidy control [35]. Similarly, experimental disruption of SPC24 impairs K–MT attachment stability and reduces the generation of euploid mouse oocytes [27]. Therefore, normal chromosome positioning alone does not necessarily indicate accurate attachment or euploid chromosome segregation. Human studies provide the greatest clinical relevance, although they are constrained by limited availability of material and the difficulty of combining live spindle imaging with direct chromosome analysis [9,11,12,13,34]. Collectively, these studies support the concept that unstable spindle assembly, abnormal spindle pole organisation, and defective chromosome orientation are plausible direct contributors to human oocyte aneuploidy. Stronger evidence for causality comes from animal models, in which spindle components, cortical signals, and cell-cycle regulators can be manipulated directly. Studies examining spindle displacement, microtubule organisation, AURK function, and cohesin-associated pathways show that perturbations in spindle regulation can lead to chromosome misalignment, chromosome segregation errors, and the formation of aneuploid oocytes [22,31,33,35,36,37,38,39,40,41,42].

## 4. Maternal Age and Oocyte Aneuploidy

### 4.1. Aneuploidy and the U-Shaped Relationship with Advanced Maternal Age

**Figure 3:**
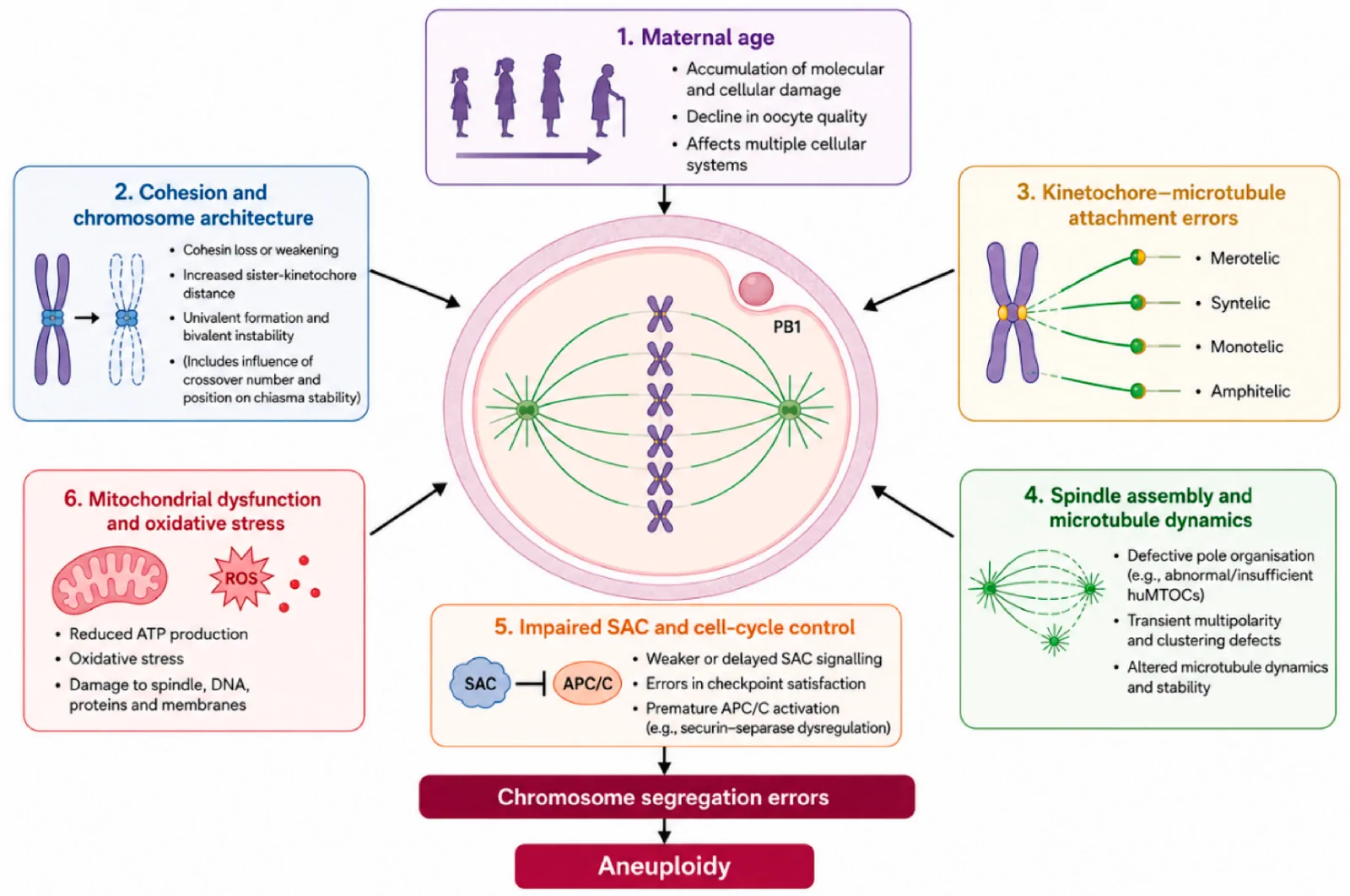
Major mechanisms contributing to chromosome segregation errors and aneuploidy in human oocytes. APC/C: anaphase-promoting complex/cyclosome; DNA: deoxyribonucleic acid; huMTOC: human microtubule-organizing center; PB1: first polar body; ROS: reactive oxygen species; SAC: spindle assembly checkpoint.

The relationship between maternal age and oocyte aneuploidy is best described as U-shaped [43]. An interesting study by Gruhn and colleagues investigated how chromosome errors in human oocytes change across the female reproductive lifespan and whether these errors could help explain the decline in natural fertility at both very young and advanced maternal ages. The researchers combined two independent cohorts: oocytes from females aged 9.1–38.8 years, obtained from ovarian tissue, and oocytes from women undergoing gonadotrophin-stimulated IVF aged 20–43. Together, these cohorts allowed the researchers to examine chromosome segregation across an unusually broad age range, from childhood through AMA. Aneuploidy rates were elevated in both very young females and women of AMA, although these increases appeared to arise from different meiotic errors. In very young females, particularly those younger than 20 years, there was an increased frequency of whole-chromosome non-disjunction during meiosis I (MI-NDJ), in which homologous chromosomes fail to separate properly. Interestingly, the frequency of this error decreased with increasing female age, suggesting that the elevated aneuploidy observed in young females is caused by a mechanism distinct from that responsible for age-related aneuploidy later in life. In older women, the predominant problems were different and largely involved the loss of chromosome cohesion. Precocious separation of sister chromatids (PSSC) increased progressively with age, while reverse segregation (RS) increased particularly from middle reproductive age into the AMA group. These errors were associated with progressive weakening of the structures that maintain sister-chromatid cohesion. The biological basis of increased MI-NDJ in younger cohorts of women (9–19 years) remains largely unknown, indicating that maternal age influences chromosome segregation through distinct pathways at different stages of the reproductive lifespan [4]. In this context, Ottolini and colleagues investigated how meiotic recombination influences chromosome segregation in human oocytes and early embryos [43]. The authors mapped thousands of maternal and paternal crossovers and identified patterns of chromosome segregation. The study revealed an unusual form of RS, as well as evidence of chromosomal drive during meiosis II. Importantly, they found evidence of selection for higher maternal recombination rates, as oocytes with lower recombination rates were more frequently associated with aneuploid embryos. Overall, the findings demonstrate that recombination plays a critical role and influences chromosome segregation and embryo viability [43]. Nevertheless, AMA remains the strongest established clinical risk factor for oocyte aneuploidy [13,44,45,46]. Rather than reflecting a single molecular defect, maternal ageing progressively compromises several systems that must remain functional throughout the prolonged prophase I arrest of human oocytes. These include chromosome architecture, sister-chromatid cohesion, spindle assembly, K-MT attachment correction, SAC signalling, mitochondrial function, and protein homeostasis. Spindle abnormalities and aneuploidy should therefore be viewed as parallel outcomes of shared upstream deterioration rather than as a simple sequence in which an abnormal spindle invariably causes aneuploidy (Figure 3). Maintenance of chromosome architecture is particularly age-sensitive. Cohesin complexes are loaded before meiotic arrest and must preserve sister-chromatid cohesion and bivalent integrity for decades. Progressive cohesin loss destabilises chiasmata, promotes dissociation of bivalents into univalents, and increases the distance between sister kinetochores. These changes increase the probability of meiosis-I-inappropriate attachment geometries, including independent attachment of sister kinetochores to opposite spindle poles [13,14,47]. Recombination further contributes to this vulnerability: the number and position of crossovers determine chiasma stability, and chromosomes with few or unfavorable position crossovers may be particularly susceptible to age-related cohesion loss and nondisjunction features. Human oocyte studies suggest that chromosome morphology influences susceptibility to attachment errors as cohesion declines, indicating that intrinsic chromosome characteristics modulate the consequences of ageing rather than conferring chromosome-specific vulnerability [48,49]. Ageing may also impair the surveillance pathways that prevent chromosome segregation before attachment errors are corrected. Reduced abundance or kinetochore recruitment of SAC proteins may shorten the time available for error correction while permitting premature APC/C activation [13,14,16,18]. Prolonged metaphase II (MII) arrest, delayed fertilisation, or extended in-vitro culture can independently impair spindle stability and checkpoint function. In mice, post-ovulatory ageing destabilises SAC activity and increases meiosis II aneuploidy, suggesting that chromosome segregation fidelity may also be influenced during the final stages of oocyte maturation and prolonged handling [50]. Ageing additionally affects spindle mechanics and microtubule dynamics. The acentrosomal MS relies on continuous microtubule turnover, motor-protein activity, and efficient correction of erroneous K-MT attachments. Age-related alterations in tubulin post-translational modifications, cortical signalling, or spindle organisation may increase the persistence of incorrect attachments despite preserved spindle morphology. In mouse oocytes, premature cortical CDC42 signalling increased microtubule tyrosination, destabilised kinetochore fibres, and increased aneuploidy, while dysregulated tubulin acetylation has similarly been linked to defective spindle organisation and chromosome alignment [31,51].

### 4.2. Relationship Between Mitochondrial Activity and Reactive Oxygen Species

Mitochondrial dysfunction, oxidative stress, and declining proteostasis likely act as broader upstream contributors. Spindle assembly and chromosome congression are energy-intensive processes, whereas reactive oxygen species (ROS) damage proteins involved in microtubule regulation and chromosome maintenance. Age-related mitochondrial dysfunction may therefore reduce ATP availability while increasing oxidative damage [52,53]. Declining protein homeostasis may further compromise cohesin-associated proteins, SAC components, spindle regulators, and mitochondrial function. In aged mouse oocytes, AURK B deficiency was associated with oxidative stress, reduced SAC-protein abundance, and impaired checkpoint activity, although a decline in proteostasis remains a plausible upstream contributor rather than an established primary cause of age-related meiotic defects in human oocytes [22]. Overall, maternal age influences chromosome segregation through multiple interacting cellular and molecular pathways rather than through a single dominant mechanism. Although the underlying mechanisms contributing to increased aneuploidy may differ between very young and AMA, both age extremes appear to converge on disruption of key chromosome architecture and cohesion, spindle function, and meiotic surveillance. Advanced maternal age represents the major cause of this network-level decline, reflecting the progressive deterioration of mechanisms that maintain the correct chromosome segregation in human oocytes. Nevertheless, investigating the mechanisms underlying the U-shaped relationship between maternal age and aneuploidy may provide broader insights into how chromosome segregation is regulated across the reproductive lifespan and how distinct age-related perturbations ultimately produce a common outcome of increased chromosomal instability. The major cellular and molecular mechanisms contributing to chromosome segregation errors and oocyte aneuploidy are summarized in Table 2.

## 5. Spindle Visualization and Its Clinical Relevance in ART

The association between MS integrity and aneuploidy has stimulated interest in non-invasive assessment of oocyte quality during ART cycles. Polarised-light microscopy enables visualisation of birefringent microtubule bundles in living MII oocytes [32,33]. This technique has been explored to assess spindle presence, position, and morphology prior to intracytoplasmic sperm injection (ICSI). A visible, centrally positioned, and morphologically normal spindle may indicate completion of nuclear maturation and assembly of an organised microtubule structure, making spindle imaging an attractive adjunctive marker of oocyte competence [10,24]. Direct visualisation of the spindle allows embryologists to choose the safest injection angle and minimise potential iatrogenic spindle injury during sperm injection [54]. Despite these advantages, spindle imaging has not become a routine method for selecting euploid oocytes. The absence of a visible spindle is not a clear indication of spindle dysfunction, as it may result from spindle orientation relative to the optical axis, timing of assessment, limited birefringence, or transient remodelling during MII. Conversely, the presence of a normal bipolar spindle does not confirm correct K-MT attachment, intact sister-chromatid cohesion, or euploid chromosome content. Important abnormalities, including merotelic attachments, altered sister-kinetochore geometry, and residual cohesion loss, may remain undetectable despite apparently normal MS morphology [10,12,13,14]. Accordingly, the relationship between MS morphology and chromosome aneuploidy should currently be regarded primarily as a topic of basic and translational research rather than a clinical decision-making tool. Although spindle abnormalities have been associated with reduced developmental competence in several studies, there is no evidence that spindle morphology should determine whether a mature oocyte can be fertilised. Consistent with current European Society of Human Reproduction (ESHRE)/Alpha recommendations, all mature MII oocytes retrieved during a fresh ART cycle should be inseminated regardless of spindle visibility or morphology [55,56]. Clinical studies linking spindle morphology with fertilisation, embryo quality, blastocyst formation, or pregnancy outcomes should also be interpreted cautiously. These outcomes are influenced by numerous factors, including sperm quality, fertilisation, cytokinesis of blastomeres, culture conditions and selection criteria. Although spindle morphology may reflect overall oocyte competence, it cannot currently serve as a validated surrogate marker of chromosome euploidy. Whereas spindle imaging provides only indirect information about chromosomal competence, preimplantation genetic testing for aneuploidy (PGT-A) directly analyses cell DNA obtained from trophectoderm biopsy. Essentially, PGT-A detects chromosome copy-number abnormalities but provides limited information regarding their developmental origin [57,58]. More recently, single-nucleotide polymorphism (SNP) based approaches combining allele frequencies with copy-number profiles have enabled distinction between predominantly meiotic and mitotic chromosome abnormalities and, in many cases, inference of parental origin and whether errors arose during meiosis I or II without requirement for parental reference DNA [58,59]. Although these methods improve biological interpretation of aneuploidy, the clinical validity and optimal indications for PGT-A remain under evaluation [60].

### Potential Iatrogenic Harm Associated with In-Vitro Temperature Variation and Cryopreservation

**Figure 4:**
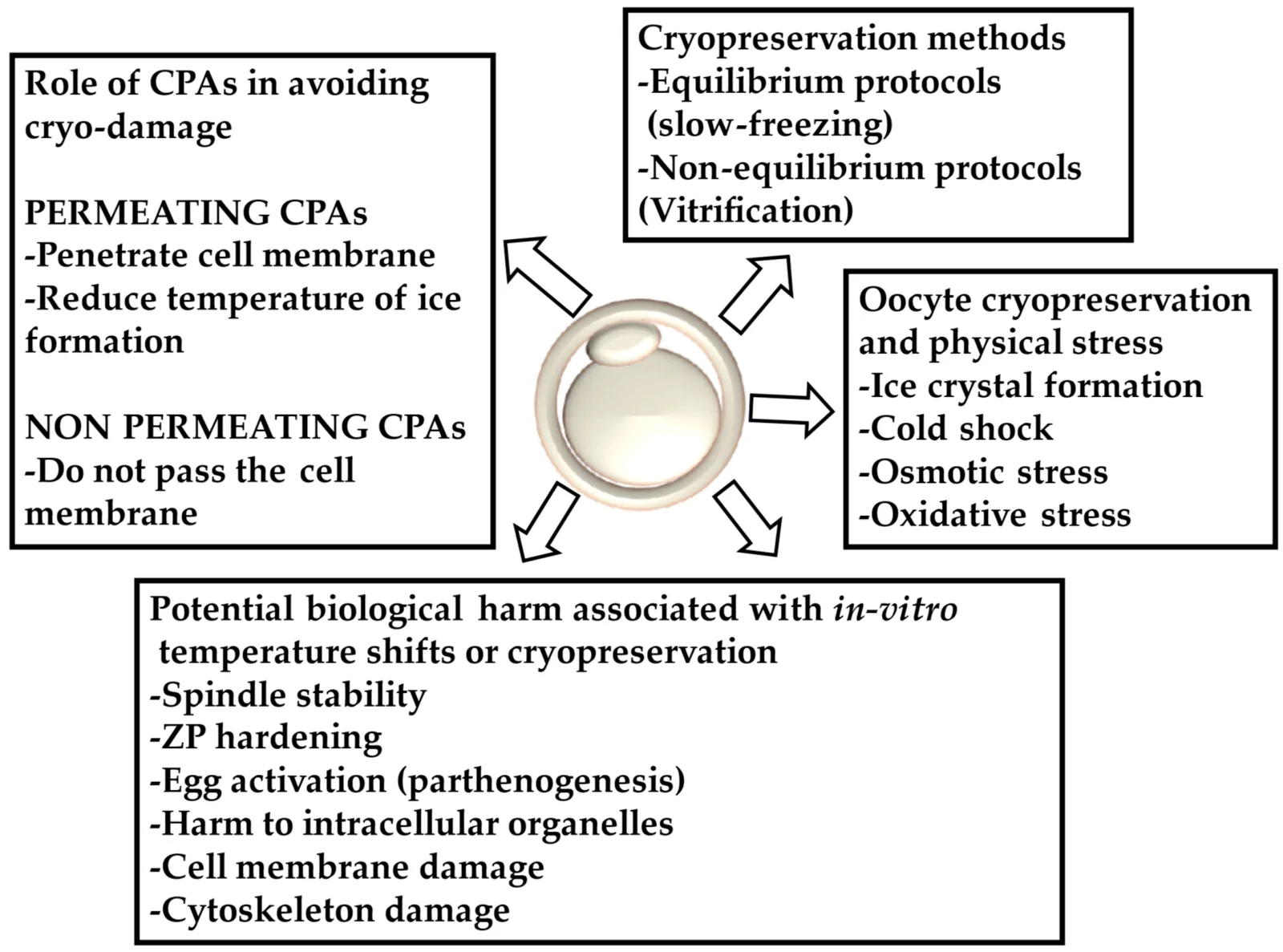
Potential physical and biological factors which may impact spindle integrity during oocyte cryopreservation. CPAs: cryoprotectant agents; ZP, zona pellucida.

Mature MII oocytes are particularly vulnerable to physical and chemical alterations during ART cycles. In particular, exposure to cooling, low temperatures and shifts in pH might adversely affect oocyte integrity and result in irreversible cellular damage (Figure 4). The susceptibility of the oocyte to environmental stress is largely attributed to its relatively large size, high intracellular water content, and distinctive cytoplasmic organization. Among these, the MS is considered particularly sensitive to changes in the surrounding environment and may therefore be susceptible to external changes or disturbances during in-vitro manipulations. Oocyte cryopreservation, including vitrification and subsequent warming, represents a critical stage during which MS integrity may be compromised. The stability of the MS is strongly influenced by temperature fluctuations, as clearly demonstrated in previous studies [61,62]. However, spindle damage and instability appear to be considerably more frequent following conventional slow-freezing procedures than after vitrification [61,63,64]. This difference is generally attributed to prolonged exposure to suboptimal temperatures and the formation of ice crystals associated with slow freezing, whereas vitrification involves rapid cooling and warming in the presence of high concentrations of cryoprotective agents, minimizing intracellular ice formation. As previously described, the MS is a highly dynamic cytoskeletal structure composed primarily of microtubules. These filaments consist of α- and β-tubulin heterodimers that interact with a range of microtubule-associated proteins (MAPs), which contribute to spindle organization, stability, and function [23,63,64,65]. Because microtubule polymerization is highly temperature-dependent, the MII spindle becomes unstable when exposed to temperatures outside the physiological range. Experimental studies have demonstrated that microtubule depolymerization may begin at approximately 33 °C and progressively increases as the temperature decreases [66,67]. Furthermore, exposure to non-physiological temperature or pH conditions for as little as 10 min has been reported to induce partial or complete spindle disassembly [66,68]. Several studies involving both animal models and human oocytes have demonstrated an association between alterations in environmental conditions, including temperature and osmolality, and abnormalities in microtubule organization and spindle morphology [66,67,68,69]. During cryopreservation, gradual exposure to cryoprotective agents may help minimize osmotic stress and preserve spindle organization. Nevertheless, spindle integrity may still be significantly affected during the warming phase, which represents a particularly critical step in the cryopreservation procedure [70,71]. Consequently, following warming, an appropriate period of in vitro culture is generally recommended to allow sufficient time for spindle reorganization and recovery before IVF or ICSI [72]. Importantly, because alterations in spindle structure have been associated with errors in chromosome segregation and embryo aneuploidy, the potential consequences of cryopreservation on spindle integrity have raised concerns regarding the genetic competence of embryos derived from cryopreserved oocytes. However, available evidence indicates that, when oocytes are optimally vitrified and warmed, cryopreservation does not appear to increase the incidence of aneuploidy in the resulting embryos [73,74].

## 6. Future Therapeutic Perspectives

Current management of age-related reproductive decline focuses largely on selecting euploid embryos using PGT-A rather than preventing the meiotic chromosome segregation errors that generate aneuploidy. However, increasing understanding of the molecular mechanisms governing oocyte meiosis has shifted attention towards interventions that preserve the cellular systems required for faithful chromosome segregation. Emerging strategies can be broadly divided into two complementary approaches: preserving the intracellular environment that supports meiotic accuracy and directly targeting the chromosome segregation machinery. Although biologically attractive, these approaches remain experimental, and none has yet been shown to prevent meiotic aneuploidy or improve live-birth rates by restoring chromosome segregation fidelity in humans. The most advanced approaches aim to maintain the intracellular environment in which meiosis occurs rather than modify individual spindle components.

### 6.1. Enhancement of Oocyte Metabolic Activity

Mitochondrial dysfunction, oxidative stress, and declining proteostasis progressively develop during oocyte ageing and may collectively impair ATP production, spindle assembly, chromosome congression, SAC function, and chromosome cohesion [46,61,62,63]. Consequently, incorporation of metabolic interventions such as coenzyme Q10 (CoQ10) and the nicotinamide adenine dinucleotide (NAD^+^) precursors nicotinamide riboside (NR) and nicotinamide mononucleotide (NMN) have been investigated to improve mitochondrial metabolism and redox homeostasis. Upon cell entry, NR is catalysed by nicotinamide riboside kinase and metabolised into NMN, which is then converted into the redox enzyme cofactor, NAD^+^, by nicotinamide mononucleotide adenylyltransferase. In aged mouse models, restoration of NAD^+^ metabolism improves mitochondrial function, reduces oxidative stress, preserves spindle organisation, and enhances developmental competence [75,76]. In the bovine model, Hashimoto and colleagues [77] investigated whether NMN supplementation could improve bovine oocyte developmental competence. They found that NMN increased intracellular NAD(H) levels, enhanced mitochondrial function and ATP production, and reduced ROS levels. NMN treatment also decreased chromosome lagging during anaphase, suggesting improved chromosome segregations. Importantly, these metabolic and cellular changes were associated with enhanced embryo developmental competence and increased blastocyst formation following IVF. The authors proposed that elevated NAD(H) levels improve post-fertilization development primarily through enhanced mitochondrial function. Furthermore, NR supplementation has been shown to restore spindle assembly and chromosome alignment in aging mouse oocytes over a period of 24 h postovulatory in-vitro culture [78]. However, human evidence remains limited, and neither CoQ10 nor NAD^+^-based therapies have been shown to reduce meiotic aneuploidy or improve live birth rates through enhanced correct chromosome segregation [46,79]. Proteostasis has also emerged as a key determinant of oocyte competence. Human oocytes remain arrested for decades and therefore rely on coordinated lysosomal, proteasomal, and mitochondrial pathways to maintain protein integrity and organelle function. Disruption of this network may simultaneously impair cohesin-associated proteins, SAC components, microtubule regulators, and mitochondrial enzymes, linking cellular ageing to chromosome separation failure [80]. Accordingly, interventions targeting mitochondrial function, redox balance, and proteostasis should be regarded as complementary strategies to preserve the cellular environment that supports meiotic accuracy rather than as direct anti-aneuploidy therapies.

### 6.2. In Vitro Maturation of Immature Oocytes

Optimising meiotic maturation represents another upstream approach. Various approaches to in-vitro maturation (IVM) of human oocytes have been applied clinically but with varying success, from so-called ‘rescue’ IVM of oocytes retrieved from follicles that apparently failed to resume their first meiotic division in response to the exogenous trigger injection of human chorionic gonadotrophin (hCG), to ‘true’ IVM where immature cumulus-enclosed germinal vesicle (GV) stage oocytes are retrieved from small follicles without prior exposure to any controlled ovarian stimulation (COS). In vivo, meiotic arrest of GV oocytes is maintained by the inhibitory environment of the follicle via gap junctions that effectively constitute a syncytium of thecal, granulosa and cumulus cells in communication with the oocyte, with arrest being overridden by administration of hCG following COS in ART cycles. A recent experimental approach to improving the maturation and developmental potential of immature oocytes from abbreviated (3–4 days) COS, with or without an hCG trigger, has incorporated co-culture of cumulus-oocyte complexes (COCs) with steroidogenic granulosa-like ovarian supporting cells derived from human-induced pluripotent stem cells [81]. That approach resulted in a significant increase in oocyte maturation rate and euploid blastocyst formation following ICSI of IVM oocytes, suggesting that paracrine factors produced by the supporting cells improved the maturation environment of the COCs. An alternative approach, biphasic capacitation-IVM (CAPA-IVM), prolongs physiological meiotic arrest in vitro through C-type natriuretic peptide-mediated maintenance of cyclic nucleotide signalling within COCs, promoting improved synchronisation of nuclear and cytoplasmic maturation with meiotic resumption [82]. Clinical studies have demonstrated the safety and reproducibility of CAPA-IVM, particularly in women with polyendocrine metabolic ovarian syndrome [83,84,85,86]. Although not intended to reverse established chromosome abnormalities, CAPA-IVM seeks to optimise the biological processes preceding spindle assembly and chromosome segregation, and its effects on meiotic fidelity remain to be established.

### 6.3. Supplementation or Replacement of Oocyte Organelles

A more invasive strategy is cytoplasmic supplementation or mitochondrial replacement. Rather than directly correcting existing chromosome abnormalities, these approaches aim to restore the functionality of the ageing oocyte cytoplasm by replenishing mitochondria and other cytoplasmic components that are essential for oocyte maturation and meiotic progression. In theory, improving the cytoplasmic environment could enhance several interconnected cellular processes, including mitochondrial function, energy production, spindle assembly and chromosome segregation. However, these approaches are unlikely to reverse chromosome segregation errors that have already been established during meiosis. A paper by Labarta and collaborators [87] investigated if autologous mitochondrial transfer could improve outcomes in patients with previously failed IVF treatment. An ovarian cortex biopsy was performed to isolate cells and their mitochondria. Sibling MII oocytes were randomly allocated to a control group or to an experimental group in which mitochondrial supplementation was achieved following injection along with the sperm during ICSI. Viable blastocysts from both groups were biopsied for PGT-A. Results reported no difference in euploid rate per biopsied blastocyst (43.8 ± 41.7% versus 63.8 ± 44.1%) nor in euploid rate per MII (9.8 ± 20.5% versus 11.9 ± 16.1%) between the mitochondrial injection group and the control one. Moreover, no differences were seen regarding mitochondrial DNA content and relevant morphokinetic variables, concluding that mitochondrial injection does not improve outcomes in the ART population [87]. Moreover, clinical application remains limited by several challenges, including technical complexity, mitochondrial heteroplasmy, the need for precise manipulation of oocytes, and unresolved ethical and regulatory considerations. Therefore, although cytoplasmic supplementation and mitochondrial replacement represent potentially promising strategies, further evidence is required to establish their safety, efficacy, and long-term reproductive outcomes [83,84,85,86,87,88].

### 6.4. Modulation of Spindle Assembly and Chromosome Cohesion

Another therapeutic strategy targets the chromosome segregation machinery directly. Maintenance of sister-chromatid cohesion, protection of centromeric cohesion, SAC signalling, and AURK-dependent error correction represent attractive therapeutic targets because age-related deterioration of these mechanisms contributes directly to chromosome mis-segregation [4,13,43,44]. However, these pathways are essential for normal meiosis progression, making their therapeutic manipulation particularly challenging. Excessive checkpoint activation or inappropriate modulation of AURK activity, cohesin dynamics, or centromeric protection could itself impair chromosome segregation and compromise oocyte quality. Consequently, effective interventions will likely need to restore or preserve physiological quality-control mechanisms rather than simply activating or inhibiting individual molecular targets. Although this field remains at an early stage, emerging preclinical evidence suggests that pharmacological interventions aimed at reducing age-related meiotic segregation errors may be feasible [89]. Collectively, these developments represent a conceptual shift in reproductive medicine, from primarily selecting euploid embryos to potentially preserving the cellular mechanisms that maintain chromosome integrity during oocyte ageing.

## 7. Conclusions

The MS is central to female meiosis because it integrates chromosome architecture, microtubule dynamics, K-MT attachment, and cell-cycle regulation into a single apparatus responsible for accurate chromosome segregation. Unlike somatic cells, human oocytes assemble the spindle in the absence of centrosomes, relying on highly coordinated microtubule self-organisation. Although this specialised mechanism enables meiosis to proceed, it also renders the oocyte particularly vulnerable to age-related disturbances. Increasing evidence indicates that defects in the MS and chromosome segregation arise from the progressive deterioration of several interconnected cellular systems. In particular, ageing is associated with loss of sister-chromatid cohesion, altered kinetochore structure, impaired spindle assembly and microtubule dynamics, defective K–MT attachments, reduced capacity for error correction, mitochondrial dysfunction, increased oxidative stress, and compromised SAC activity. These defects can act independently or synergistically, ultimately increasing the probability of chromosome mis-segregation and aneuploidy. The relationship between spindle abnormalities and aneuploidy is therefore biologically compelling, although spindle morphology alone does not necessarily reflect the chromosomal status of an oocyte. Importantly, aneuploid embryos may result from errors arising during maternal meiosis or from post-zygotic mitotic divisions, emphasising the need to distinguish these mechanisms when interpreting embryonic chromosome abnormalities. Within ART, spindle imaging provides valuable information regarding oocyte competence and has an established procedural role during ICSI; however, its ability to predict embryo euploidy remains limited. Meanwhile, advances in PGT are providing increasingly detailed information on the parental and developmental origins of aneuploidy. Overall, understanding the mechanisms that compromise meiotic fidelity during oocyte ageing may be more valuable than focusing solely on the selection of euploid embryos following fertilisation. Future reproductive strategies should therefore explore approaches aimed at preserving or restoring the molecular systems governing chromosome cohesion, kinetochore function, spindle organisation, mitochondrial activity, and cell-cycle surveillance. However, any intervention designed to modify these fundamental processes must be evaluated rigorously prior to clinical implementation, with particular emphasis not only on reproductive efficacy but also on embryo developmental potential, genomic stability, and long-term offspring health. Achieving this goal will require a deeper understanding of oocyte ageing and rigorous evaluation of emerging therapies to ensure not only efficacy but also safety.

## Figures and Tables

**Table 1:** Key differences in meiotic spindle assembly and chromosome segregation between mouse and human oocytes. K-MT: kinetochore-microtubule attachment; MT: microtubule; aMTOC: acentrosomal microtubule-organizing center; huMTOC: human microtubule-organizing center; NEBD: nuclear-envelope breakdown; SAC: spindle assembly checkpoint.

Feature	Mouse Oocyte	Human Oocyte	Key References
Centrosomal organization	Canonical centriole-containing centrosomes are absent. Multiple prominent aMTOCs provide major sites of MT nucleation and contribute to pole formation.	Canonical centriole-containing centrosomes are absent. Human oocytes lack the prominent mouse-like aMTOC system and rely more strongly on chromosome- and kinetochore-associated MT nucleation.	[9,11,21]
Initial spindle assembly	After NEBD, multiple aMTOCs fragment and redistribute around chromosomes, generating MTs that are progressively organized into the spindle.	Spindle assembly is predominantly chromosome- and kinetochore-mediated. Recent evidence suggests that a distinct huMTOC may additionally contribute to MT nucleation following NEBD.	[9,19]
Spindle bipolarization and pole formation	Fragmented aMTOCs are sorted and clustered into two spindle poles, providing relatively robust pole-organizing structures.	Kinetochore-associated MT minus ends initially coalesce into multiple minor poles, which progressively aggregate into two major poles.	[11,19]
Duration of MI spindle assembly	Typically occurs within a few hours (~3–5 h).	Exceptionally prolonged; spindle assembly and chromosome congression require approximately 16 h.	[9,10,11]
Transient multipolarity and spindle stability	Multiple MTOC-derived foci are a normal assembly intermediate, but the aMTOC system supports efficient bipolarization and relatively stable pole organization.	Pronounced transient multipolarity is a characteristic intermediate of bipolarization. Human spindle poles show greater organizational instability and may undergo splitting or defocusing.	[9,11,17]
Chromosome congression and K–MT attachment	Chromosome alignment occurs within a shorter assembly interval. Experimental perturbation of spindle positioning or K-MT regulation can increase incorrect attachments and aneuploidy.	Chromosome congression is prolonged and can be asynchronous, potentially extending the interval during which erroneous K-MT attachments may form and persist.	[9,11]
Checkpoint control and error correction	SAC and Aurora kinase-dependent error-correction pathways are active and experimentally tractable; their perturbation can cause chromosome misalignment and aneuploidy.	Core SAC components are active, although direct mechanistic evidence in human oocytes remains more limited than in the mouse. Mammalian oocyte SAC control can permit anaphase progression despite a limited number of persistent attachment defects.	[14,16,23]
Experimental relevance	Highly amenable to live imaging, genetic manipulation and targeted perturbation, allowing strong causal testing; however, the prominent aMTOC system limits direct extrapolation to humans.	Experimental access is limited, but direct live imaging has revealed a prolonged, chromosome-mediated and intrinsically error-prone spindle-assembly pathway of direct relevance to human reproduction.	[9,11]

**Table 2:** Major mechanisms contributing to chromosome segregation errors and aneuploidy in oocytes. K-MT: kinetochore-microtubule.

Mechanism	Effect on Oocyte Meiosis	Consequence for Chromosome Segregation
Cohesion loss	Destabilization of bivalents, increased sister-kinetochore separation and loss of meiosis-I-specific chromosome architecture.	Precocious separation of sister chromatids, reverse segregation and nondisjunction.
Altered kinetochore geometry	Impaired sister-kinetochore co-orientation and increased susceptibility to inappropriate microtubule capture.	Erroneous K-MT attachments and chromosome segregation errors.
Spindle instability/defective pole organization	Delayed or incomplete bipolarization, unstable pole focusing and persistent abnormal spindle architecture.	Chromosome misalignment and increased risk of mis-segregation.
Erroneous K-MT attachment	Formation or persistence of inappropriate attachment configurations, including merotelic attachments.	Lagging chromosomes and unequal chromosome segregation.
Impaired spindle assembly checkpoint	Insufficient delay of meiotic progression when attachment defects remain.	Persistence of chromosome attachment errors into anaphase.
Altered microtubule dynamics	Impaired kinetochore-fibre stability, microtubule turnover and spindle organization.	Increased attachment errors and chromosome mis-segregation.
Mitochondrial dysfunction and oxidative stress	Reduced ATP availability and oxidative damage to pathways supporting spindle assembly, chromosome congression and checkpoint function.	Reduced meiotic fidelity and increased susceptibility to segregation errors.
Post-ovulatory ageing	Progressive destabilization of spindle organization and checkpoint function during prolonged MII arrest.	Increased risk of meiosis-II chromosome segregation errors.

## Data Availability

No new data were created or analyzed in this study. Data sharing is not applicable to this article.
